# Interactive dose shaping part 1: a new paradigm for IMRT treatment planning

**DOI:** 10.1088/0031-9155/61/6/2457

**Published:** 2016-03-07

**Authors:** Peter Ziegenhein, Cornelis Ph Kamerling, Uwe Oelfke

**Affiliations:** Joint Department of Physics, The Institute of Cancer Research and The Royal Marsden NHS Foundation Trust, London, SM2 5NG, UK; Peter.Ziegenhein@icr.ac.uk; Uwe.Oelfke@icr.ac.uk

**Keywords:** treatment planning, intensity modulated radiation therapy, real-time dose calculation, interactive dose shaping

## Abstract

In this work we present a novel treatment planning technique called interactive dose shaping (IDS) to be employed for the optimization of intensity modulated radiation therapy (IMRT). IDS does not rely on a Newton-based optimization algorithm which is driven by an objective function formed of dose volume constraints on pre-segmented volumes of interest (VOIs). Our new planning technique allows for direct, interactive adaptation of localized planning features. This is realized by a dose modification and recovery (DMR) planning engine which implements a two-step approach: firstly, the desired localized plan adaptation is imposed on the current plan (modification) while secondly inevitable, undesired disturbances of the dose pattern elsewhere are compensated for automatically by the recovery module. Together with an ultra-fast dose update calculation method the DMR engine has been implemented in a newly designed 3D therapy planning system Dynaplan enabling true real-time interactive therapy planning. Here we present the underlying strategy and algorithms of the DMR based planning concept. The functionality of the IDS planning approach is demonstrated for a phantom geometry of clinical resolution and size.

## 1. Introduction

Intensity modulated radiation therapy (IMRT) planning requires the optimization of a set of parameters which determine the radiation dose to be delivered to a patient. The treatment parameters are usually found by solving an inverse problem, defined by a set of dose-volume constraints for a set of pre-segmented, organ specific volumes of interest (VOI). Conventionally the inverse problem is solved by an iterative quasi-Newton optimization method. The optimization is driven by a single or multi-criterial objective function accounting for the previously defined dose-volume constraints. The optimal plan (the optimal set of treatment parameters) is found by minimizing the objective function.

This rather indirect way of deriving a treatment plan suffers from various inherent shortcomings. First, the minimized objective function represents a mathematically optimal plan which depends on the notion of the objective function itself. It is not guaranteed that this plan is clinically optimal or even acceptable. A clinically relevant objective function inevitably comprises conflicting planning constraints. The trade-off between these constraints is acknowledged using a penalty factor which weights the importance of conflicting dose-volume constraints between the radiation target and organs at risk. However, a penalty factor has no direct clinical meaning. Its impact on the planning process cannot be assessed until the optimization process is finished. Thus, a tedious trial-and-error approach is necessary in order to find an acceptable set of planning parameters.

Second, the control of local planning features is limited to the pre-defined VOIs. The same prescribed dose and penalty factors are usually assigned to all voxels of a specific organ such that local dose features within a selected VOI cannot be controlled. Due to this restriction it is especially difficult to compensate for hot spots in the normal tissue or to explicitly shape an iso-dose line within a VOI. Third, the conventional planning method does not easily facilitate a plan adaption to a changed patient geometry.

During the last 15 years several attempts were made to overcome most of these inherent drawbacks of the Newton-based optimization method: Cotrutz and Xing (2002, 2003) developed an improved optimization method to consider a regionally variable penalty scheme. The optimization process in his work consists of two parts. First, several conventional trial-and-error optimization runs are performed to find an acceptable set of VOI specific penalty factors while all voxel specific penalty factors are set to 1. In a second step the user modifies prescribed dose pattern locally by increasing certain voxel specific penalty factors. The authors demonstrate that some degree of local control can be brought to the planning process. However, varying a voxel specific penalty scheme is a tedious, time consuming process. Furthermore it is not guaranteed that a local change of voxel specific penalty factors does not influence desired features of the plan elsewhere. A similar approach was proposed by Lougovski *et al* (2010) where an automated procedure was introduced to vary the prescribed dose in each voxel.

Another user friendly planning approach called multi-criterial-optimization (MCO) was suggested by Thieke *et al* (2007). MCO is based on a pre-optimized set of treatment plans which are pareto optimal for the considered set of dose constraints. An MCO-based planning framework provides an interactive interface which allows the user to navigate along the pareto-surface. However, MCO is based on a pre-optimized set of plans which has to be recalculated as soon as the patient anatomy changes which makes fast adaptive planning difficult. An attempt to overcome this problem of MCO was introduced by Süss *et al* (2013). It offers a decision-making tool to continuously change a plan between an almost acceptable anchor plan and one or more alternative plans which have a specific local insufficiency removed. Although this method provides an interactive plan alteration it is still based on the formulation of an objective function. It provides a framework to select between different plans but it does not allow a direct interaction with the dose distribution of the plan. An interactive treatment planning technique has been recently introduced by Otto (2014). He discusses a pre-optimization method which allows for direct interaction with the dose distribution of the plan. The so achieved desired features are then translated into dose-volume constraints and exported to a conventional plan optimization. The pre-optimization step works on a reduced dose grid employing a simplified pencil beam algorithm which allows for instant dose calculations within a few milliseconds. The fast dose engine is one of the key features enabling the interactivity of the presented pre-optimization method. Using simplifications in the dose algorithm is feasible in this step since the actual deliverable plan is created in the subsequent optimization.

In this work we introduce an alternative and new approach for therapy planning called interactive dose shaping (IDS). IDS does not employ a conventional optimization to derive a plan. It allows for a direct manipulation of local planning features such as shaping an isodose surface or manipulating dose of individual voxels without compromising already established planning goals elsewhere. A plan is found as a result of a sequence of such local feature adaptations which are controlled interactively by the planner. The local plan modifications can be performed in real-time so that the planner gets an immediate response when a certain planning goal is imposed. This real-time feature of IDS is achieved by extensive use of modern parallel computing techniques.

## 2. Methods and materials

### 2.1. The IDS concept

**Figure 1.**
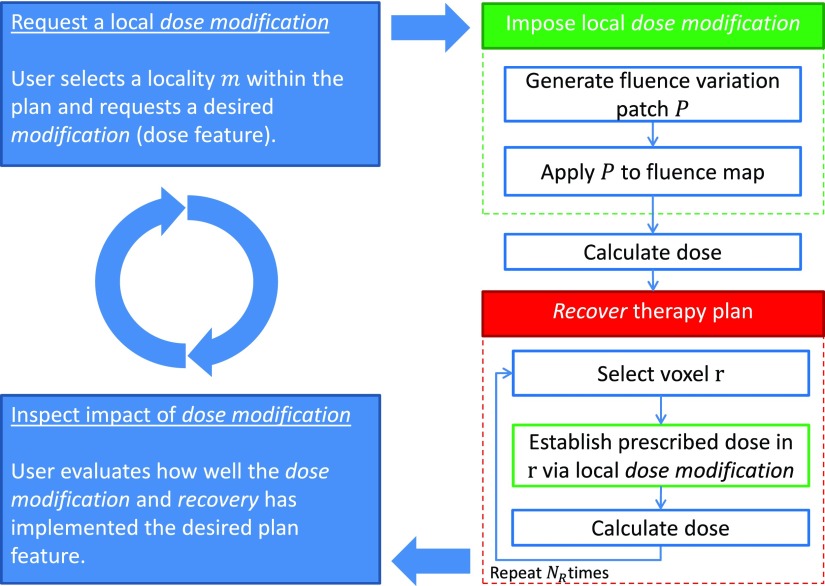
Workflow of the IDS planning technique. The user (top blue box) formulates a localized desired planning feature which is imposed by the modification module (green box). Locality of the modification is enforced by the recovery module (red box) which aims to restore the dose in voxels r where an original dose value was compromised. The user gets a real-time response (lower blue box) and requests another dose modification.

The IDS planning technique aims to directly modify localized features in a therapy plan without compromising already established features elsewhere in the plan. In order to accomplish that, IDS relies on a two-step *dose modification and recovery* (DMR) approach whose basic idea is illustrated in figure 1.

The dose modification step (green box in figure 1) imposes a local plan adaptation in location *m* as requested by the planner. An adaptation such as a local modification of an isodose surface or a dose value modification in one voxel is formulated by the planner directly via an interactive 3D graphical user interface provided by a TPS. The modification algorithm described in section 2.2 adds a fluence patch *P* to each incident beam to facilitate the requested change. This alteration of the fluence map will not only implement the requested local dose modification but will inevitably compromise established and desired dose features in other locations of the plan. In order to restrict the dose modification to the desired location *m*, the recovery step (red box) described in section 2.3 aims to compensate for unintentional changes elsewhere in the plan while preserving the initially requested dose modification as far as possible.

A major challenge for the development of IDS is the required realization of a real-time response during the planning process via the graphical interface. The respective DMR process requires dose up-dates within a few milliseconds, which is accomplished by an ultra-fast dose calculation strategy described in section 2.4.

In the flowing three sections 2.2–2.4 we will describe and illustrate the basic components of our IDS framework before first results for a horseshoe shaped target phantom are presented in section 3.

### 2.2. The dose modification module

The key element of our dose modification strategy is the exploitation of all available prior knowledge about the patient-geometry, the fixed set-up of the incident beam directions and the dosimetric characteristics of the radiation beam. This combined knowledge will be accumulated in a set of heuristic metrics and rules guiding the whole dose modification and dose recovery process.

Naturally, there is no unique way of harvesting this information but any set of guiding principles sufficiently reflecting the physical boundaries of achievable dose patterns will lead to a successful planning strategy. Another recently published example for these heuristic local planning concepts is the work by Otto (2014). In contrast to these new methods, conventional inverse planning concepts analyse the same prior information during the optimization of a non-unique objective function based on dose volume constraints of entire VOIs and therefore loose the flexibility to impose local dose features.

**Figure 2.**
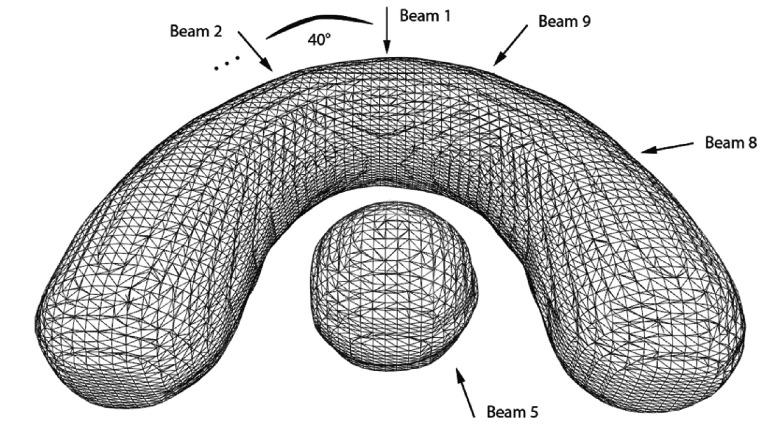
3D mesh representation of the phantom used to demonstrate the dose modification and recovery strategy. The horse-shoe shaped target is irradiated by nine equispaced beams which are arranged counterclockwise around the phantom.

**Figure 3.**
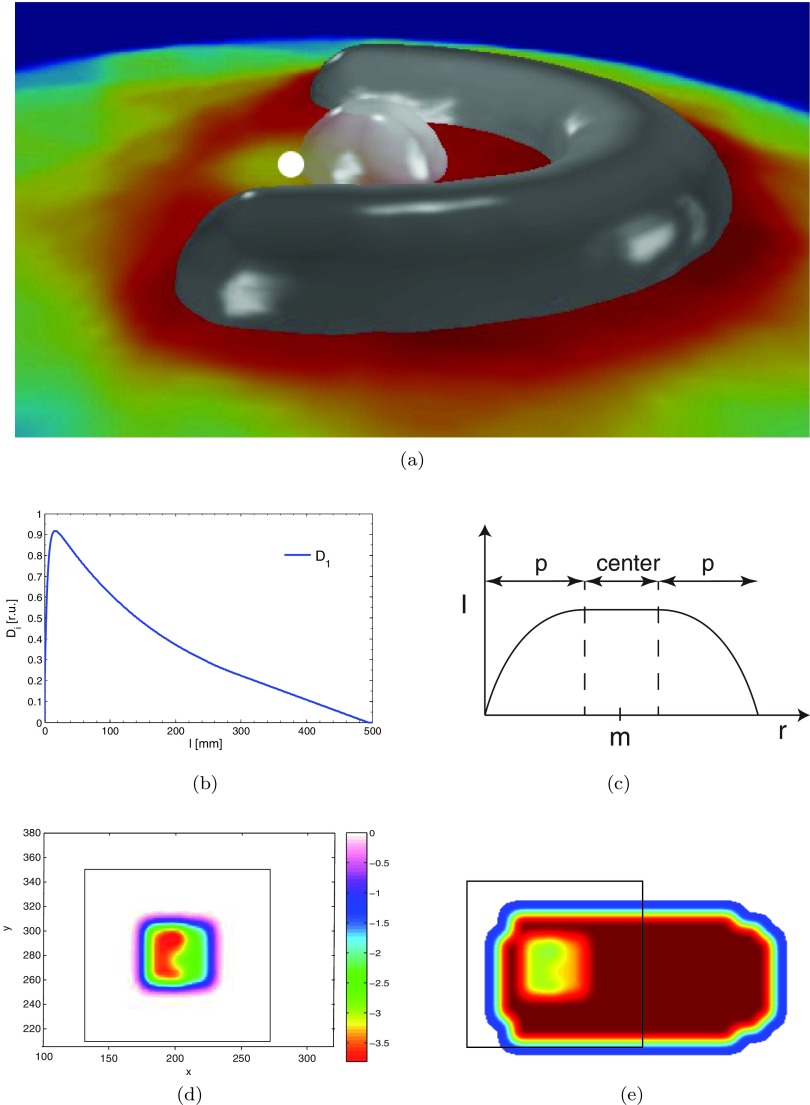
Dose modification example. (a) Tilted beams-eye view of the phantom geometry as seen from beam 8. (b) Depth dose curve on the central axis. (c) Kernel *K*(*x*, *y*) describing the projection of the lateral dose kernel from *m* onto the fluence map. (d) Modification patch. (e) Fluence map of beam 8 after modification patch has been applied.

Dose modifications in our approach are enforced in a hierarchical order starting with the most elementary request of changing the dose within a single voxel. This basic component is then applied to achieve localized dose changes for selected groups of voxels as defined by 2D-isodose-lines or 3D-isodose-surfaces. In the following we will consider in detail the modification of the dose within a single voxel and illustrate our algorithms in the context of the classical IMRT phantom geometry consisting of a horseshoe shaped target encompassing a spherical organ at risk as displayed in figure 2. Resolution and size of the phantom geometry is of clinical quality: the voxel resolution is $1.952\times 1.952\times 2$ mm^3^ while the diameter of the horse-shoe is about 12 cm. The planning setup consists of 9 equally spaced coplanar beams. In order to demonstrate the dose modification strategy a request to change the dose value in only one voxel *m* (marked as white spot in figure 3(a)) was made. The initial plan prior to the modification consists of an open field arrangement that homogeneously fills the target volume with dose.

As an example we consider lowering the dose to the voxel *m* indicated in figure 3(a) from its original value *d*0 to *dt*. How much should we lower the value of each individual fluence amplitude contributing to the dose in this point? In order to answer this question, we first identify for each beam *b* lateral regions of interest in fluence space with a central representative coordinate *s*(*x*, *y*). Each control point *s*(*x*, *y*) represents $5\times 5$ fluence amplitudes of 0.5 mm radius. Next we calculate heuristic measures of the relative dosimetric importance of these amplitudes for the two relevant tissue types tumour targets (*T*) and organ at risks (*O*). This is achieved by defining a traveling path information *TPI*^*T*, *O*^ which accumulates the doses *D*_1_ of the depth dose curve at radiological depth *l*(*i*) of voxel *i* which is delivered to either tumour or OAR by a specific amplitude of beam *b*,

1 \begin{eqnarray*}TP{{I}^{T,O}}\left(b,{{s}_{x,y}}\right)=\underset{i\in {{V}^{T,O}}\left(b,{{s}_{x,y}}\right)}{\sum}\,{{D}_{1}}\left(l(i)\right)\end{eqnarray*}

i.e. it contains the explicit knowledge about its relevant radiological path length, the incident beam direction and its depth dose curve as displayed in figure 3(b). As a final metric we account for the lateral penumbra of the fluence amplitudes by a lateral projection of the dose kernel *K*(*x*, *y*) centred around the voxel *m* as shown in figure 3(c). *K*(*x*, *y*) is hereby derived from the second lateral pencil beam kernel component *w*_2_ introduced in equation (5).

Having defined these metrics, we next specify the rules of how they are to be used for the realization of a local dose modification. The guiding principle of our approach is that our local dose modification should keep any anticipated adverse effect in competing tissues small. Lowering doses in healthy tissues or organs at risk is expected to lower doses to the tumour target and the expected size of this effect therefore determines which fluence amplitude is reduced by what amount. For our example illustrated in figure 3 this is accomplished by assigning a weighting factor:

2 \begin{eqnarray*}A{{(b,x,y)}^{T,O}}=K(x,y)\exp \left(-\mu \times TP{{I}^{T,O}}\left(b,{{s}_{x,y}}\right)\right)\end{eqnarray*}

to each amplitude where *K*(*x*, *y*) characterizes the lateral importance of the fluence amplitude and *TPI*^*T*^ reflects the anticipated unwanted dose reduction for the tumour target. The attenuation coefficient *μ* is chosen so that the exponential factor of equation (2) is 0.5 for the mean TPI value over all contributing beams of one control point:

3 \begin{eqnarray*}\exp \left(-\mu \underset{b=1}{\overset{n}{\sum}}\,\frac{TP{{I}^{T,O}}\left(b,{{s}_{x,y}}\right)}{n}\right)\overset{!}{{=}}\,0.5\end{eqnarray*}

The relative weighting factors *A* are projected onto the fluence maps of each beam direction and are finally multiplied with a global scaling factor to ensure that the target dose to voxel *m* is lowered from *d*0 to *dt*. Figure 3(e) shows the derived fluence patch of weighting factors *A* for beam 8 in our considered example. Appendix provides the pseudo code of how the local dose modifications are implemented. A corresponding rule applies to the increase of local target doses and their impact on OARs.

### 2.3. The dose recovery module

**Figure 4.**
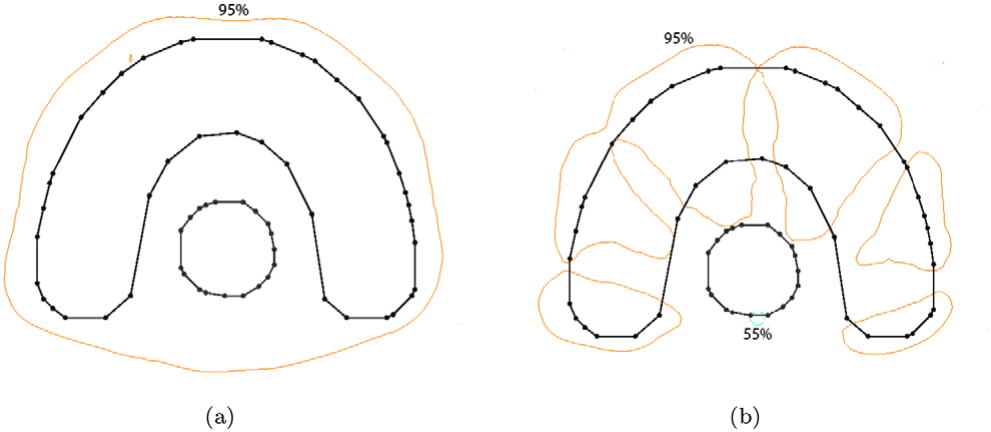
Isodose lines (55%, 95% and 107%) on a slice of the phantom geometry. (a) Before the modification process. (b) After the dose modification without recovery.

The dose modification module as described in the last section aims to impose the desired dose alteration to voxel *m*. The concept of the TPI scoring map was used to keep the disturbance of the anticipated dose pattern outside of *m* small. However, any local dose modification comes at the price of unavoidable, unwanted dose changes to dose competing structures. In our example, shown in figure 4(b), the reduction of dose in voxel *m* naturally creates cold spots in the tumour target along the lines where the modified fluence amplitudes intersect the tumour. In order to reach our goal of a localized dose modification around *m* we have to *recover* the dose in these areas by involving a set of different fluence amplitudes. This task is facilitated by the dose recovery module.

The dose recovery process is largely based on the same principles as the dose modification process. Any local cold spot in the target is scored with the same TPI driven metric (equation (1)) just that now inverse local dose modifications based on *TPI*^*O*^ are facilitated such that the dose values prior to the dose reduction in *m* are restored. However, the recovery process is a bit more complex. First, there exist whole groups of target voxels whose dose has to be restored to different initial dose levels, i.e. one needs to define a strategy in which order these modifications should be facilitated. Second, these dose recovery steps can be in conflict with the original local dose modification and may require to slightly changing the dose value achieved at the initial dose modification process.

Both problems are addressed by the following strategy. For each voxel required to be involved in the recovery process we first determine its geometrical distance from the initial point of the requested dose modification. This distance map is calculated by the distance transform technique of (Borgefors 1984). This map determines the priority of which voxels are restored first.

In our approach we pursue a *close in* dose recovery approach by restoring first the dose in voxels that are located furthest away from our original dose modification at voxel *m*. This strategy has two advantages. First, the dose differences to be restored initially are likely to be small such that the required amplitudes changes are small and second, there is less probability that we have to compromise our originally achieved dose modification. However, within this process we are slowly closing in on the sensitive conflict area of dose gradients established by the local dose change in *m*. In this area of dose gradients the recovery will finally probe the physical possible compromise between the initial dose modification and the inflicted local changes in its adjacent areas of interest. Previously performed dose recovery processes in locations further away usually alleviate the problem of not compromising the initial dose request.

**Figure 5.**
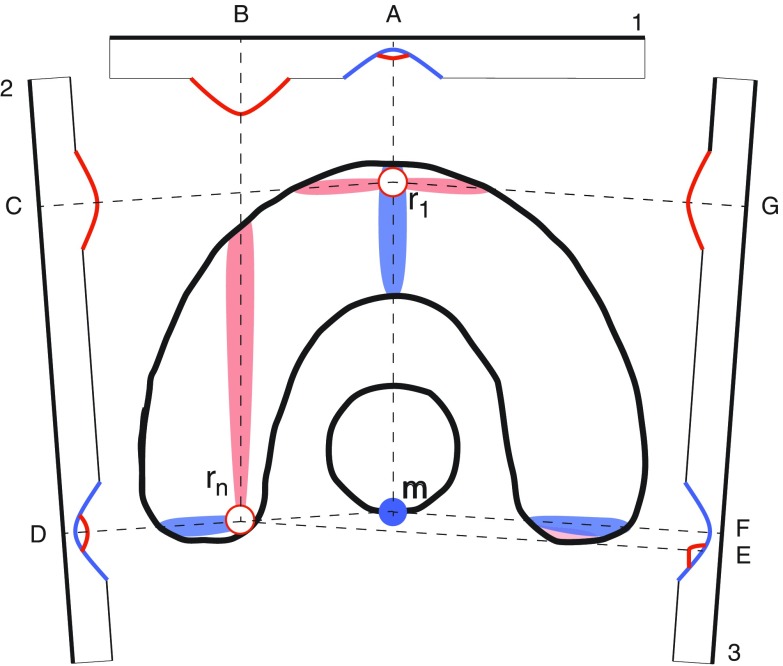
Selection of the recovery points and determination of the beam angle contribution on the phantom geometry after imposing a modification on *m*. Only three out of nine beams are shown for the sake of clarity.

In the following we will discuss and illustrate our dose recovery strategy for the chosen phantom geometry and consider for the sake of clarity only three initial beam directions in a 2-dimensional projection as indicated in figure 5. The initially requested dose modification in *m* is shown at the bottom of the OAR as a cold spot (blue circle). The dose modification in *m* has been imposed by applying beam fluence variation patches in the projection of *m* shown as blue sections of the beam intensity around point A, D and F. These modifications result in the indicated blue areas of target cold spots which are subject to the dose recovery process described above. According to the selection process introduced earlier the recovery module chooses the location *r*_1_ as the first spot to be recovered. *r*_1_ projects to location A,C and G on the respective beams where patches resulting in an increase of fluence amplitudes have to be applied. The intensity of patches in C and G are almost equally high while the intensity in A is chosen to be lower, resulting from the TPI scoring map. While amplitudes around C and G only influence target voxels, an increase of intensity in A has a negative effect on the initially requested dose modification in the OAR. Nevertheless, location A is not neglected from the process. The DMR strategy allows small compromising recovery patches to the disadvantage of the initial modification. This is necessary to guarantee the convergence of the planning process. The recovery strategy will try to compensate the compromising fluence modification in the next DMR step using more suitable beam directions. After this first recovery step is finished the dose pattern is updated and it could be shown that the initial dose around *r*_1_ could be re-established (white circle).

Figure 5 also shows the recovery of a second cold spot around *r*_*n*_. *r*_*n*_ is located closely to *m* and therefore selected later in the recovery process. It projects to fluence amplitudes around B,D and E. However, fluence amplitudes in D and E have also been used to impose the initial requested dose feature in *m* so dose modification and dose recovery patches overlap in these regions. The TPI scoring map accounts for this conflict and allocates a larger intensity to the patch in B while recovery patches in D and E are applied with a significantly lower intensity. Due to the recovery process itself the target homogeneity is also disturbed by second-degree hot spots which form in the projection pathways of the recovery locations (red areas of the target). However the absolute dose deviance of these hot spots is small compared to the unwanted cold spots which have been created due to the initial dose feature request. Nevertheless these second-degree deviations are also subject to the recovery process and can be selected based on there location and absolute dose deviance.

Usually a dose modification request triggers the recovery of 15–30 locations within competing tissue to compensate for unwanted dose changes outside the selected area. After the recovery process is finished the resulting dose distribution is presented to the planner. The trade-off between imposing the requested dose modification and re-establishing the dose pattern outside of the feature area can be directly assessed.

### 2.4. Ultra-fast dose update calculation

A complete update of the dose pattern is necessary after each modification and recovery patch has been applied to assess the effect of the respective operation on the treatment plan. Since several recovery steps are needed for imposing one local dose modification request, the dose calculation has to be performed in a time frame of less than one second in order to preserve the real-time character of IDS. Thus a new method designed for ultra-fast dose update calculations was developed and is introduced briefly in this section.

Starting point of our calculation is the pencil beam algorithm by Bortfeld *et al* (1993) based on a singular-value decomposition:

4 \begin{eqnarray*}D_{\text{irreg}}^{\prime}\left({{x}_{p}},{{y}_{p}},l\right)=\underset{i=1}{\overset{3}{\sum}}\,D_{i}^{\prime}(l){{C}_{i}}\left({{x}_{p}},{{y}_{p}}\right)\end{eqnarray*}

5 \begin{eqnarray*}{{C}_{i}}\left({{x}_{p}},{{y}_{p}}\right)=\underset{x}{\sum}\,\underset{y}{\sum}\,\psi (x,y){{w}_{i}}\left(x-{{x}_{p}},y-{{y}_{p}}\right)\end{eqnarray*}

which calculates the dose $D_{\text{irreg}}^{\prime}\left({{x}_{p}},{{y}_{p}},l\right)$ at a point characterized by a radiological depth *l* and lateral isocentric coordinates $\left({{x}_{p}},{{y}_{p}}\right)$ which is caused by an irregularly shaped and spatially modulated fluence pattern $\psi (x,y)$ also defined in the isocentric plane. $D_{i}^{\prime}(l)$ and ${{w}_{i}}\left(x-{{x}_{p}},y-{{y}_{p}}\right)$ represent the depth dependent weighting factors and the lateral dose kernels derived by the singular value decomposition.

Despite its exceptional speed this algorithm in its original implementation is not fast enough to facilitate real-time interactive treatment planning. Even a more recent implementation on modern parallel hardware architectures (Siggel *et al* 2012) still fails to provide the required calculation speed.

However, we were able to accelerate the dose calculation to the desired speed by the following measures. First, we aimed to exploit the limited spatial extent of the fluence patches which modify the doses in each interactive step of the planning process. A restriction of the dose update to the modified fluence amplitudes, however, can only be achieved by performing the convolution of equation (5) in coordinate space without switching to Fourier Space and therefore cannot exploit the performance of fast Hartley transforms.

**Figure 6.**
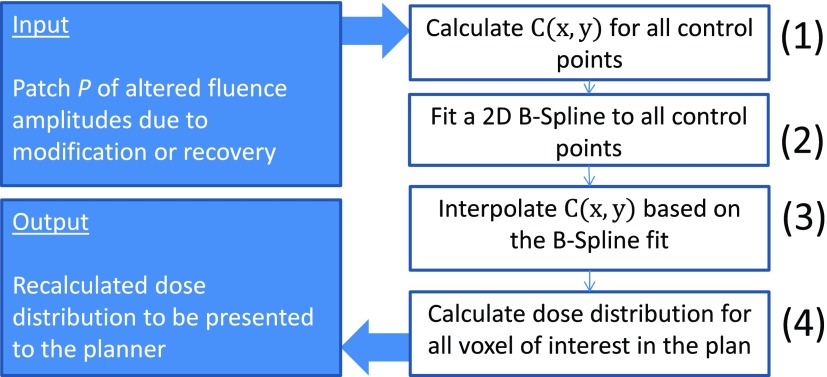
Workflow of the fast dose update calculation in IDS.

To speed up the calculation even further, the convolution in equation (5) is only calculated for a set of evenly distributed control points and then interpolated for all other points of interest. The workflow of this process is shown in figure 6. The dose calculation starts automatically immediately after the modification or recovery module proposed a patch *P* of altered fluence amplitudes.

## 3. Results

### 3.1. Dose modification and recovery on a phantom

**Figure 7.**
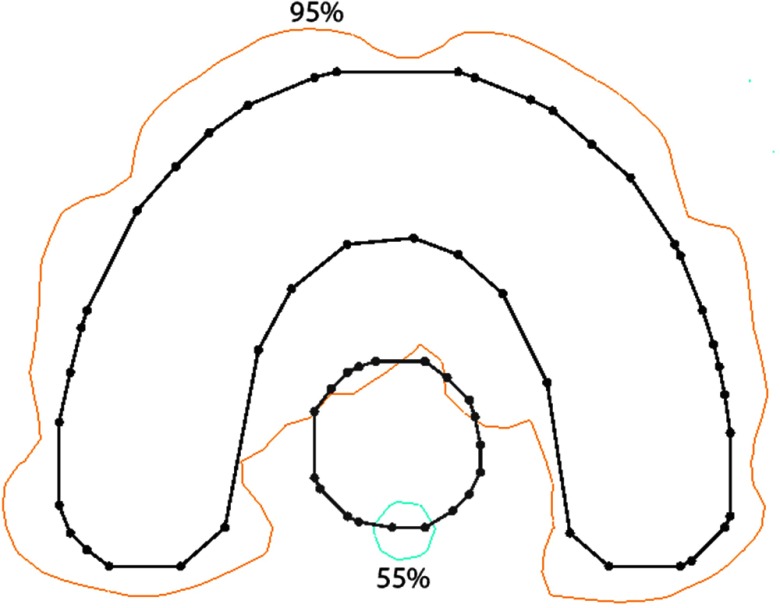
Isodose lines (55%, 95% and 107%) after 25 recovery steps subsequent to the modification in *m*.

The dose distribution of the horse-shoe shaped phantom plan after the recovery process was finished is shown in figure 7. By comparing it to the distribution prior to the recovery process (figure 4(b)) one notices that the dose in the target area could be restored almost completely. All target voxels show a dose higher than 95% of the prescribed dose while the dose at the rim of the OAR was lowered to 55%. In addition figure 7 reveals that the intended cold spot around the initial variation location has become larger. This is due to the nature of continuous energy transport in space and the requested dose feature itself is also subject to the recovery process. Thus, the recovery not only aims to restore the initial target dose but also tries to preserve the dose features requested. In this case the recovery module chooses to expand the requested dose feature to neighboring voxels because there are no other constraints preventing it. This result was achieved by applying 25 recovery steps subsequently to imposing the initial dose request. The runtime for modification and recovery including graphical representation of the result in 3D was about 5 s.

### 3.2. IDS planning framework

In order to develop and evaluate the IDS strategies we designed a completely new TPS for interactive planning. The presented IDS strategies and the dose update algorithm have been carefully implemented with regard to modern CPU architectures. The result is a fast realization of the IDS workflow. The runtimes of the key components used within the DMR module are listed in table 1 measured on the phantom geometry presented in figure 2. Selecting a new location on which the recovery should be carried out takes approximately 40 ms for a plan of clinical size and resolution. This includes the evaluation of the effect of the previous recovery step and the selection of the best candidate spot according to the strategy described in section 2.3. The time for creating the actual modification patch is comparably low since it is a straight forward task which only involves a small amount of data. The convolution of the pencil beam kernels as described in section 2.4 takes in the order of 10 ms for all three kernels of all 9 beam directions. The most time of approximately 60 ms is consumed by looping through the dose cube and applying the updated dose value to every voxel in the plan. In addition table 1 also reveals the runtimes for creating the TPI map over the range of a patch area and the runtime for calculating the distance grid for the sake of completeness. These two key operations only have to be performed once for every patient since the TPI map and the distance map do not change for a given geometry. Another key element of our TPS is its interactive 3D graphical user interface which allows to formulate local dose modification requests in the form of selecting a voxel of the plan and change its dose, dragging an iso-dose line on a 2D plane or directly shaping an iso-dose surface in 3D. A description of this framework can be found in Kamerling *et al* (2014).

**Table 1.** Runtimes in ms for the central algorithms involved in the DMR process measured on the phantom geometry presented in figure 2.

Select location	Create patch	Calculate convolution	Update dose cube	Create TPI map	Calculate distance grid
40	<1	10	60	45	89

## 4. Discussion

In the previous sections, we have introduced a new paradigm of performing treatment planning for IMRT. Our motivation to abandon the established approach of IMRT optimization was to address inherent shortcomings of the objective function based treatment planning strategy.

First, objective function based treatment planning is a very indirect, non-intuitive way of shaping a dose distribution. The aim of the treatment planning process is to select from an enormous ensemble of treatment parameters one set that will shape the dose distribution for a given patient anatomy, such that an optimal treatment outcome can be realized within the limitations imposed by the physics of radiation transport for a certain dose delivery technology.

Conventionally, this is achieved by expressing dose constraints for pre-defined volumes of interest in mathematical form, multiplying these individual terms with a penalty factor, summing them up to establish an objective function and finally minimizing this function to derive the optimal treatment parameters. Neither does this function know anything about the patient geometry nor does it reflect any prior knowledge about the physics of radiation transport. That is why the objective function as a wish list of desirable or non-desirable dose features has to be adapted manually in the treatment planning process and why the impact of these adaptations on the dose is often hard to predict. Interactive dose shaping potentially avoids these problems because prior knowledge about the patient anatomy and dose profiles is the information employed to drive the described dose modification and recovery cycle.

Second, conventional IMRT planning struggles with the lack of tools to control local dose features. The successful planning of clinical cases with complex patient geometries often requires the creation of additional VOIs such that certain anatomical regions of the patient can be spared from or supplied with extra dose. Starting with the process of local dose variations on a voxel level as the most elemental planning action, as proposed in section 2, aims to fully control any local dose feature.

The presented IDS concept aims to realize a therapy plan by imposing a series of user specified, local dose modifications via the DMR strategy which allows for a real-time user interaction. The key functionality of the DMR strategy has been introduced in detail in this paper. The working principle has been demonstrated successfully for a 3D phantom with clinical resolution and size. We could show in a recent study (Kamerling *et al* 2016) that the implemented planning concept can be used to design treatment plans of acceptable quality for clinical cases.

The successful realization of IDS planning strategy could only be achieved by a highly efficient implementation of ultra fast algorithms within the DMR module. One DMR step including one dose modification request and dose recovery at up to 30 other dose points can be completed within only a few seconds. Due to this real-time response the user can directly evaluate the effect of a dose request and has the possibility to revise his request if the outcome is unsatisfactory. 15 to 30 recovery steps per DMR process are usually enough to implement the dose modification while restoring the rest of the planning features. However, the number of recovery steps can be set manually by the planner if he feels that the results are unsatisfying. It is also possible to trigger an extra recovery run between the dose modification steps if needed. One of the biggest challenges was the design of the dose calculation algorithm in section 2.4 which provides an ultra-low latency dose update of only a few milliseconds. The dose engine operates on fluence maps. Thus, it is necessary to run a sequencer at the end of the planning process in order to create deliverable plans.

A method similar to our outlined DMR planning strategy was recently described by Otto (2014). The pre-optimization step presented by Otto (2014) is similar to our DMR planning strategy. However, Otto (2014) still requires an additional conventional optimization step to finalize the treatment planning process while IDS generates plans without a subsequent optimization. In order to do that, our DMR algorithm employs a dose calculation method and plan generating strategies which are both highly accurate and very fast.

## 5. Conclusion

In this paper we have described the general principle and a first realization of a new IMRT treatment planning strategy based on interactive dose shaping. Naturally, this development is still at the beginning and currently cannot provide all the sophisticated functionality provided by a conventional IMRT planning system, e.g. features like direct aperture optimization or the use of very accurate dose algorithms accounting for tissues inhomogeneities are not available in its current stage. However, we believe that the outlined treatment planning strategy bears the potential for the development of interesting tools and applications for clinical IMRT planning ranging from interactive treatment plan refinement of pre-optimized plans to major plan modifications required by changing patient anatomies.

## Appendix IDS algorithms

**Algorithm 1.** Generation of a modification patch

1:	**function** createPatch (voxel *i*, TPIMap *T*, Kernel *K*, targetDose *d*_*t*_)	
	**input:** = voxel *i*, the voxel which has been selected for modification or recovery	
	TPIMap *T*, TPI scoring for each beam	
	Kernel *K*, lateral beam characteristic (identical for each beam)	
	targetDose *d*_*t*_, the requested dose in voxel *i*	
	**output:** $P$, set of fluence Manipulation patch for each beam	
	**local variables:** = $\#beams$, number of beams	
	*A*, normalized temporary patch	
	*k*, lateral kernel profile at (*x*, *y*) relative to the central axis	
	*t*, TPI value for amplitude (*x*, *y*) of one specific beam	
	*μ* attenuation factor according to equation (3)	
	*f*, scalar factor specifying the intensity of the fluence amplitude manipulations	
2:	$d0\leftarrow doseAt(i)$	$\triangleright $ save original dose in voxel *i*
3:	**for** $b=1..\#beams$ **do**	$\triangleright $ create normalized patch
4:	$A(b).box\leftarrow boundingBox(b,i)$	$\triangleright $ geometrical limits of the patch
5:	**for all** $(x,y)\in A.box$ **do**	
6:	$t\leftarrow T(b,x,y)$	
7:	$k\leftarrow K(x,y)$	
8:	$A(b,x,y)\leftarrow k\times \exp (-\mu t)$	$\triangleright $ according to equation (2)
9:	**end for**	
10:	**end for**	
11:	$d1\leftarrow doseCalc(i,A)$	$\triangleright $ re-calculate dose only in voxel *i* given patch A
12:	$f\leftarrow |{{d}_{t}}-d0|\div |{{d}_{t}}-d1|$	$\triangleright $ determine patch scaling
13:	$\Delta P\leftarrow A\otimes f$	$\triangleright $ $\Delta P$ holds a modification patch for each beam
14:	**end function**	
